# Supplementary material for: Learning curve and surgical outcome of robotic assisted colorectal surgery with ERAS program
Source: Sci Rep. 2022 Nov 29;12:20566. doi: 10.1038/s41598-022-24665-w (PMC9709162; doi:10.1038/s41598-022-24665-w)
Supplement: Supplementary file 2 — Supplementary Information 2. [file 41598_2022_24665_MOESM2_ESM.zip › RAL Raw data-Ñ[▒K/055/OP N_ñw╝╨░O▒Kñσ.pdf]

W95 / W95

臺中榮民總醫院
手術室護理記錄

[redacted]

基本資料

- 室別： 開刀房-17室，手術科別： CRS，手術類別： 常規，日期： 2021/01/23
- 進入時間： 0735 ； 麻醉開始時間： 0805 ； Time Out時間： 0910 ； 劃刀時間： 0911 ； 手術結束時間： １７２５ 送出時間： １７５０ ；
- 麻醉方式： GE

  診斷：Malignant tumor of rectum

  手術名稱：Robot Assisted Low Anterior Resection

  手術局部用藥：NIL.

  術前預防性抗生素：
  - 抗生素名稱一： Veterin 1GM IVD由麻醉護理師執行
  - 時間： 0908
  - 上次簽章: 邱文琇

  ，追加預防性抗生素：
- 手術醫師： 蔣鋒帆，陳映菁，李一宏
- 麻醉醫師： VS 蔡志忠
-
  - 刷手護士A ： 朱芸萱，時間： 0735，刷手護士B ： 陳家瑋，時間： 0735，接替刷手一： 邱曉玲，時間： 1300，接替刷手二： 劉裴瑀，時間： 1500
  - 流動護士A ： 邱文琇，時間： 0735，接替流動一： 陳宥廷，時間： 1200，接替流動二： 張秀敏，時間： 1500

皮膚狀況

A-V Shunt： 無

術前： 完整

記錄者： 上次簽章: 邱文琇

術後： 完整

記錄者： 張秀敏

手術準備

手術臥式 單一手術臥式

-
  - 手術臥式： 截石臥式
  - 使用附件： 溫毯 43 ℃，頭圈，腳架，其它： lateral Support、海綿、頭架
  - 皮膚準備： 10%Aq Betadine，Hibiscrub，2% Chlorhexidine
  - 電燒： 使用電燒: 電極片位置： 右大腿，雙極電燒，記錄者： 上次簽章: 邱文琇

止血帶T： 無

植入物

無

標本送檢

- 細菌培養 無
- 細胞學 無
- 冰凍切片 無
- 病理標本

  組織名稱一：para aortic LN

  ,

  組織名稱二：IMA ROOT

  ,

  組織名稱三：distal cut end

  ，更多組織名稱
  - 組織名稱四：rectum

- 截肢 無

標本交付 標本： 4 袋 ， 標本： 0 盒 ， 標本： 0 瓶 ， 細胞學： 0 管 ， Culture Tube： 0 管 ， 被交付醫師： CR 陳映菁，

- 記錄者： 上次簽章: 張秀敏
- 時間： 1600

傷口術後敷料

傷口敷料：

- 請於圖上標示部位：

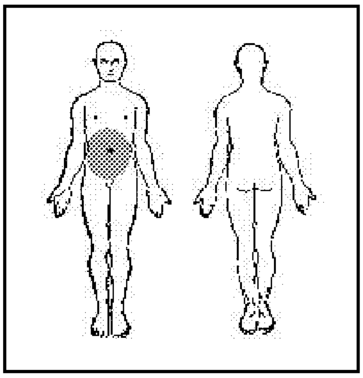

- 傷口敷料： 紗布

導管

導管	天數	導管資訊	插入日期	插入時間	更換日期	拔除日期	拔除時間	拔除原因	預計拔除/更換	預計拔除/更換日期
Epidural PCA(1)	1	部位:硬脊膜外;	2021/01/23	0810						
A-line (1)	1	號碼:20 G; 部位:左手臂;	2021/01/23	0835						
CVC(1)	1	號碼:8 Fr.; 部位:; 象限:RU; 註記:fix 15cm;	2021/01/23	0840						
Foley(1)	1	號碼:16 Fr.; 部位:膀胱;	2021/01/23	0845						

用物計數

- 手術中出血量： minimal c.c.
- 手術中尿量： １６５０ c.c.
-
    - 其它： 無

用物計數： 多切口同時縫合

- 1.器械： 正確 ，2.縫針： 正確 ，3.刀片 ： 正確 ，4.紗布： 正確 ，5.腹部墊： 未使用 ，6.紗球： 未使用 ，7.PEANUTS ： 未使用 ，8.紗條： 未使用 ，9.絲帶： 未使用 ，10.針頭： 未使用 ，11.Vessel loop： 未使用 ，12.Boots： 未使用 ，13.Jelco： 未使用 ，14.棉條： 未使用 ，
    - 第一次：
        - 刷手護士 ： 劉裴瑀
        - 流動護士： 上次簽章: 張秀敏
        - 時間： 1545

    ,
    - 第二次：
        - 刷手護士 ： 劉裴瑀
        - 流動護士： 上次簽章: 張秀敏
        - 時間： 1550

    ,
    - 第三次：
        - 刷手護士 ： 劉裴瑀
        - 流動護士： 上次簽章: 張秀敏
        - 時間： 1555

備註

備註：davinci 30度 scope NO.1

- 記錄者： 上次簽章: 邱文琇
- 時間： 0735

填寫護理人員 張秀敏

正式摘要

W95 / W95

doctorRank:B
